# Trypanosomatids Detected in the Invasive Avian Parasite *Philornis downsi* (Diptera: Muscidae) in the Galapagos Islands

**DOI:** 10.3390/insects11070422

**Published:** 2020-07-09

**Authors:** Courtney L. Pike, María Piedad Lincango, Charlotte E. Causton, Patricia G. Parker

**Affiliations:** 1 Department of Biology, University of Missouri-St. Louis, 1 University Blvd., St. Louis, MO 63121, USA; pparker@umsl.edu; 2 Charles Darwin Research Station, Charles Darwin Foundation, Santa Cruz, Galapagos Islands 200350, Ecuador; mplincangoc@uce.edu.ec (M.P.L.); charlotte.causton@fcdarwin.org.ec (C.E.C.); 3 Facultad De Ciencias Agrícolas, Universidad Central Del Ecuador, Quito 170521, Ecuador; 4 WildCare Institute, Saint Louis Zoo, One Government Drive, St. Louis, MO 63110, USA

**Keywords:** invasive insects, parasites, trypanosomatids, *Crithidia*, *Blastocrithdia*

## Abstract

Alien insect species may present a multifaceted threat to ecosystems into which they are introduced. In addition to the direct damage they may cause, they may also bring novel diseases and parasites and/or have the capacity to vector microorganisms that are already established in the ecosystem and are causing harm. Damage caused by ectoparasitic larvae of the invasive fly, *Philornis* *downsi* (Dodge and Aitken) to nestlings of endemic birds in the Galapagos Islands is well documented, but nothing is known about whether this fly is itself associated with parasites or pathogens. In this study, diagnostic molecular methods indicated the presence of insect trypanosomatids in *P*. *downsi*; to our knowledge, this is the first record of insect trypanosomatids associated with *Philornis* species. Phylogenetic estimates and evolutionary distances indicate these species are most closely related to the *Crithidia* and *Blastocrithidia* genera, which are not currently reported in the Galapagos Islands. The prevalence of trypanosomatids indicates either *P*. *downsi* arrived with its own parasites or that it is a highly suitable host for trypanosomatids already found in the Galapagos Islands, or both. We recommend further studies to determine the origin of the trypanosomatid infections to better evaluate threats to endemic fauna of the Galapagos Islands.

## 1. Introduction

Worldwide, parasites and disease are recognized threats to wildlife health [1,2,3,4]. Novel parasites and diseases may especially threaten species in island systems, which are often lacking in strategies to deal with alien species introductions [5,6,7,8]. Pathogens or parasites can be either introduced with their hosts or vectors or as a free-living stage and, once established, are often transmitted to other introduced or native species that act as new hosts or vectors thus facilitating dispersal and invasion success [8,9]. In some cases, these introduced parasites may themselves be vectors of other parasites or disease [10,11].

*Philornis downsi* (Dodge and Aitken), a known dipteran parasite of passerine birds in the Neotropics [12], was first recorded in the Galapagos Islands in the 1960s [13]. This fly has become invasive, successfully colonizing at least 15 islands in the Galapagos archipelago, bringing serious consequences to the native avian fauna due to the blood-feeding behavior of its ectoparasitic larvae [14,15,16]. While many studies have been conducted on the fly’s effects on birds e.g., [17,18], studies on its associations with parasites and pathogens have only recently been initiated. Research is now underway to study the microbiota and viruses associated with *P*. *downsi* e.g., [19], but to date nothing is known about whether this introduced fly is associated with endoparasites, including trypanosomatids.

Trypanosomatids have been reported in over 500 insect species [20]; however, it has been suggested that the number of currently reported insect hosts is just a fraction of the total host species that exist [21]. Infected insect hosts include species in the orders of Diptera, Hymenoptera, Siphonaptera, Hemiptera, and Lepidoptera, among others [22,23]. Trypanosomatids can be transmitted vertically or horizontally between insects [24,25,26,27,28], and are easily transferred via ingestion of infected fecal matter from other insects and other food sources [24,29]. Infections with trypanosomatid parasites in insects have a wide range of pathogenicity and virulence, from unnoticeable effects on their hosts to decreased energy levels, lower fitness, negative immune system effects, and sometimes death [29,30,31,32,33,34]. On the other hand, insects can act as vectors of trypanosomes that seriously affect the wellbeing of other wildlife (including mammals) or humans [35,36,37,38].

*Philornis downsi* has the potential to be a transporter of trypanosomatids that either traveled with flies from mainland Ecuador or that have adopted this invasive fly as a host since its arrival. In Galapagos, there is one report of an insect trypanosomatid, *Strigomonas culicis,* found in a single native mosquito *Aedes taeniorhynchus* [39] and reports of a *Trypanosoma* species associated with an endemic hawk, *Buteo galapagoensis* and a mosquito, *Culex quinquefasciatus* [2,39]. However, given that trypanosomatids are common and widespread throughout the world [40], it is likely that there are additional species in the Galapagos archipelago that have the potential to be picked up by *P. downsi*. Alternatively, given the broad distribution and host range of many monoxenous trypanosomatid species that infect insects [25,33,41,42], it is highly possible that trypanosomatids were introduced with *P. downsi*. If this is the case, these could pose a potential risk for the insect fauna in Galapagos.

As part of a larger study to identify parasites associated with *P. downsi* and evaluate the possible threat posed to native and endemic species in the Galapagos Islands, we screened adult *P. downsi* flies for the presence of insect trypanosomatid parasites. This research provides the first evidence of *P*. *downsi* as a transporter of endoparasites in the Galapagos Islands.

## 2. Materials and Methods

**Figure 1:**
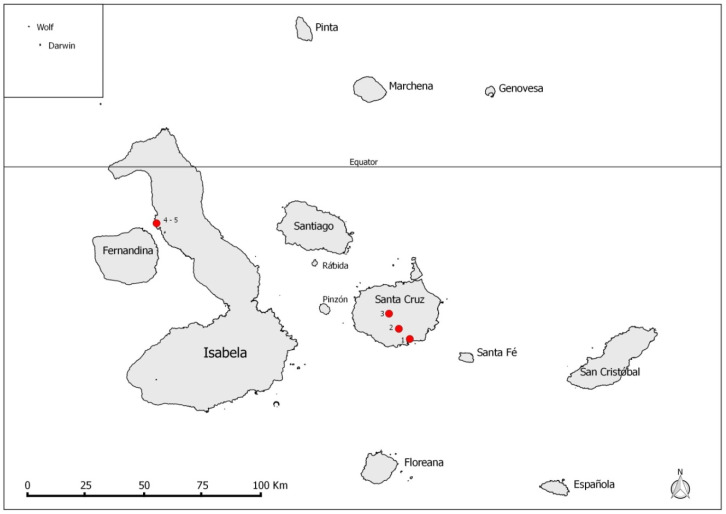
Map of the Galapagos Islands. Collection sites for *Philornis downsi* samples included (1) El Barranco (lowland dry zone), (2) Los Guayabillos (mid-elevation agricultural zone), and (3) Los Gemelos (endemic *Scalesia* forest, highland humid zone) on Santa Cruz Island, and (4) coastal mangrove habitat and (5) lava field on Isabela Island.

Sampling took place on Santa Cruz and Isabela islands in the Galapagos Archipelago (Figure 1). Both islands have human settlements. Collections of *P. downsi* occurred at low (15–41 m above sea level), mid (209–216 m), and high (589–616 m) elevations on Santa Cruz; these occurred in El Barranco—dry zone (0°44′34.1″ S, 90°18′10.4″ W), Los Guayabillos—agricultural zone (0°41′68.7″ S, 90°20′78.6″ W), and Los Gemelos—Scalesia forest, humid zone (0°37′82.0″ S, 90°23′44.4″ W), respectively (Figure 1). On Isabela Island, collections were carried out in a coastal mangrove forest, habitat of the Critically Endangered Mangrove Finch, *Camarhynchus heliobates*, at Playa Tortuga Negra (0°14′32.09″ S, 91°23′10.97″ W), and on a neighboring field of lava (0°14′46.69″ S, 91°23′5.64″ W) (Figure 1).

Collection of *P*. *downsi* specimens took place between 23 March and 26 July 2013 on Santa Cruz Island and 29 June to 4 July 2013 on Isabela Island. McPhail traps (Naturquim, Ecuador) were used to trap flies using a fresh papaya juice/sugar mix as an attractant following the recipe used by Causton et al. [43]. Thirty traps were placed at each of the three collection sites on Santa Cruz and the mangrove site on Isabela. In addition, 24 traps were placed on the lava field on Isabela. On Santa Cruz, traps were hung in trees three to four meters above ground level and *P*. *downsi* adult flies were collected every five to seven days. On Isabela, traps were hung at five, seven, and ten meters high in the mangrove forest and at two meters high on the lava field; live and dead *P*. *downsi* adults were collected within ten days of trapping. All flies were placed in 95% ethanol in 1.5 mL Eppendorf tubes. Only flies caught in the same trap were pooled in an individual sample tube, with a range of one to seven flies per tube. In total, 393 sample tubes containing 913 flies were collected from sites on Santa Cruz and Isabela.

Flies were analyzed at the University of Missouri-St. Louis. The flies from each sample tube were dried out for 12–16 h (overnight) to ensure all ethanol evaporated before extraction. Each sample analyzed contained tissue from one to seven flies. Sample tubes containing more fly tissue than was needed for proper extraction were separated into two tubes before DNA extraction, yielding a final count of 401 sample tubes (913 flies) for extraction. DNA was extracted from each sample using the QIAGEN DNeasy Blood and Tissue Kit (QIAGEN, Valencia, CA, USA). After extraction, samples were individually read on a Nanodrop spectrophotometer at wavelengths of 260 and 280 nm (nanometers) to determine the concentration of DNA. As DNA extraction resulted in strong dominance by fly DNA versus pathogen DNA and samples with overall low concentrations might lead to false negatives in pathogen testing, samples with 80 ng (nanograms)/µL(microliter) DNA or less were not used for further testing. Likewise, we excluded samples that were heavily degraded. In total, 297 samples (694 flies) showed little degradation and were used for polymerase chain reaction (PCR) testing (Table 1).

Molecular screenings were conducted using both single and nested polymerase chain reaction (PCR) tests to determine the prevalence of protozoa in the Trypanosomatidae family. These PCR tests amplify fragments in the conserved region of the small subunit (SSU) rRNA (ribosomal ribonucleic acid) gene of the parasite, a gene commonly targeted for trypanosome detection and identification [44,45,46,47]. We tested a total of 297 sample pools (Table 1) for initial trypanosomatid testing by conducting a nested PCR reaction. The initial outer PCR reaction used primers S-762 (5′-GACTTTTGCTTCCTCTA(A/T)TG-3′) and S-763 (5′-CATATGCTTGTTTCAAGGAC-3′) and the second, inner PCR reaction used primers S-755 (5′-CTACGAACCCTTTAACAGCA-3′) and S-823 (5′-CGAA(T/C)AACTGC(C/T)CTATCAGC-3′) [44]. For further trypanosomatid identification requiring longer fragments for sequencing, a subset of 95 samples, representing all localities and both sexes, were tested with primer set R221 (5′-GGTTCCTTCCTGATTTACG-3′) and Medlin B (5′-TGATCCTTCTGCAGGTTCACCTAC-3′) [45]. Prevalence data reported are based on sample pools containing up to seven flies, not on individual flies.

The first PCR conducted on all samples was a nested reaction (Table S1) that amplified a 326 b.p. (base pair) fragment [46]. The primers used from [44] in this nested PCR have been previously used to detect trypanosomes in a variety of species, including from avian, mosquito, and amphibian hosts [44,46,48], including the *Trypanosoma* detected in the Galapagos Hawk [2]. Thermal cycling conditions for the outer and inner reactions followed the conditions in [46]; however, for the 35 cycles in the inner reaction, 72 °C was run for 50 sec instead of 30 sec. Culex mosquito samples reliably testing positive for parasites of genera *Strigomonas* and *Trypanosoma* using the same primer sets were used for initial PCR testing. After initial PCR tests, *P*. *downsi* samples reliably testing positive for Trypanosomatidae parasites were also used as positive controls. After a PCR test was completed, the amplicons were run on a 1.5% agarose gel, and samples with bands near 326 b.p. were scored positive for Trypanosomatidae.

The second PCR test for Trypanosomatidae protozoa (S1) was conducted using primers from [45] testing a small subset of samples that scored positive for trypanosomatids using the [44] primer sets. This PCR test was a single reaction that targets a 1300–1400 b.p. fragment [45] targeting the 18S rRNA gene. The thermal cycling conditions for this PCR reaction included a denaturation phase of 94 °C for 3 min, followed by 30 cycles of 94 °C for 30 sec, 55 °C for 30 sec, and 72 °C for 2 min. The final elongation phase was 72 °C for 10 min. These amplicons were also run on a 2% agarose gel and positive amplicons with bright bands were saved for sequencing. *Philornis downsi* samples that reliably tested positive for Trypanosomatidae parasites using the first PCR assays were used as positive controls.

Reagents and mix amounts for Trypanosomatidae protozoa PCR tests are listed in Table S1. In addition, negative controls were included in each PCR reaction, using ddH2O (deionized distilled water) in place of genomic DNA.

Amplicons testing positive for Trypanosomatidae were sequenced. PCR amplicons scored as positive for trypanosomatids from [44,45] primer sets were purified using an Exonuclease I (Exo I) and Antarctic Phosphatase reaction (New England BioLabs Inc., Ipswich, MA, USA). Next, primers used for each PCR test were used in subsequent sequencing reactions with the Big Dye^®^ Terminator v3.1 Cycle Sequencing Kit (Applied Biosystems, Foster City, CA, USA). PCR sequencing products were cleaned up using an ethanol/ethylenediaminetetraacetic acid (EDTA)/sodium acetate precipitation. Products resuspended in Hi-Di™ Formamide (Applied Biosystems) were sequenced in both directions on an ABI 3130xl genetic analyzer (Applied Biosystems) at the University of Missouri-St. Louis. Sequences were assembled and edited in Seqman Pro 8.0.2 (DNAStar, Lasergene). Next, sequencing results were entered into NCBI BLAST (Basic Local Alignment Search Tool) for parasite identification.

Initially, a subset of samples testing positive by PCR using [44] primers were sequenced. Ten amplicons from PCR tests using R-221 and Medlin B primers [45] were also sequenced for more specific parasite identification.

The Chi-squared test with Yates’ continuity correction was used to test for differences in infection counts between the islands of Santa Cruz and Isabela and between El Barranco and Los Gemelos collection sites within Santa Cruz for sample pools tested using the [44] primer. Fisher exact tests were run for other pairwise comparisons between sites, using sample pools, except for the Los Guayabillos collection site, Santa Cruz and the lava field collection site, Isabela, due to small sample sizes. Minimum infection rates (MIR) were calculated with a weighted average using the equation:

(1) $$MIR=\left[\;\frac{number\;of\;positive\;sample\;pools}{total\;number\;of\;flies\;tested}\;\right]\times 100\%$$

A multiple sequence alignment was created in MEGA 6 [49] which included the most reliable parasite sequences acquired from *P*. *downsi* using the [45] primer set and 13 known sequences of species from the Trypanosomatidae infecting insects and birds, obtained from GenBank (Table S2). We aligned sequences using Clustal W and manually trimmed and edited the alignment to a final alignment of 1033 b.p., of which 271 sites were parsimony informative. Evolutionary nucleotide distances were calculated in MEGA 6 with 1000 replicates. A model of best fit for DNA was determined by the program and a maximum likelihood tree estimate was constructed using the K2 + G model [50] with 1000 bootstrap replicates. The tree was rooted with species from the *Trypanosoma* genus that infect avian hosts, including *T*. *bennetti*, *T*. *avium*, *T*. *corvi*, and *T*. *culicavium*. Bayesian posterior probabilities were calculated using BEAST v1.10 [51]. A GTR + G + I model of evolution was determined the best-fit model using bModeltest [52]. Ten million trees were sampled, using a burn-in of the first 10% trees. Parameters included a relaxed clock, uncorrelated lognormal and a Yule prior for speciation. A final tree was created in TreeGraph2 [53], merging Maximum Likelihood (ML) tree topology with support values from both the ML and Bayesian analyses.

## 3. Results

### 3.1. Molecular Testing

For both PCR tests, negative controls reliably appeared negative, without bands. Positive controls consistently displayed bands near the targeted fragment size of 326 b.p. using primers from [44] and within the 1300–1400 b.p. range using the primer set from [45]. Overall, the sensitivity of primer sets for trypanosomatid detection yielded differences in prevalence values. The tests that detected fragmented parasite DNA < 1100 b.p. [45] yielded lower prevalence values compared to the initial tests using the primers from [44]; however, most samples still contained fragments large enough for the primer set of [44] to detect (Table 1).

Out of 297 sample pools (694 flies total) tested using primers from [44], 267 sample pools were positive, indicating a prevalence of infected sample pools of 89%. Parasites were present in samples from all months of collection (March–July 2013) and from all collection sites. For collection sites on Santa Cruz, prevalence was 84% at the lowland, arid site (El Barranco, *n* = 165 sample pools), 99% at the humid highland site (Los Gemelos, *n* = 110 sample pools), and 100% in the agricultural zone (*n* = 2 sample pools). Trypanosomatid prevalence on Isabela Island was 82% in the mangrove forest (*n* = 17 sample pools) and 100% on the lava field (*n* = 3 sample pools). Of the 95 sample pools tested using primers from [45], the prevalence of positive sample pools was 42% (*n* = 40 sample pools). Minimum infection rates (MIR) for each site are listed in Table 1. The overall weighted MIR for all sites was 38.5%.

The prevalence of infected sample pools collected from the humid highland site was significantly higher than for the dry lowland site (X2 = 15.59, df = 1, *p* < 0.001) on Santa Cruz Island. Parasite infection prevalence was significantly higher for the humid highland site on Santa Cruz Island than for the mangrove site on Isabela Island (*p* < 0.01, Fisher’s exact test).

### 3.2. Phylogenetic Analysis

**Figure 2:**
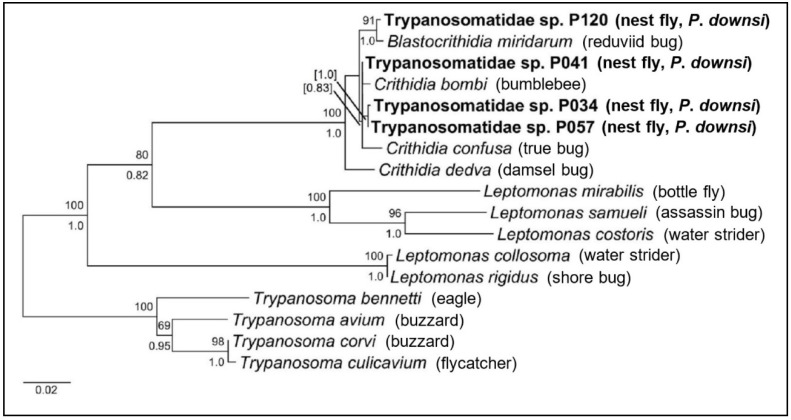
A Maximum Likelihood (ML) phylogenetic tree estimate of trypanosomatid parasite sequences derived from the 18S rRNA gene. The final alignment, deposited in [54], includes 1033 b.p, of which 271 sites were parsimony informative. Sequences from our infected *Philornis downsi* samples are indicated in bold. The tree was rooted with species from the *Trypanosoma* genus that infect avian hosts. Support values above 80% are displayed, with bootstrap values listed above nodes and Bayesian inferred posterior probabilities listed below nodes. The number of site substitutions are indicated by tree branch lengths. Accession numbers for all sequences used are listed in Table S3.

Sequences of parasites from positive sample pools were deposited in GenBank with accession numbers MG787532-MG787541 (Table S2). Groups of new parasite sequences isolated from *P. downsi* flies trapped at different localities and islands appeared identical in MEGA 6 after alignment and trimming. Some isolates from the highland and lowland collection sites on Santa Cruz appeared identical, including Trypanosomatidae sp. isolates P034 and P091, and Trypanosomatidae sp. isolates P120, P129, P322, and P361. Additionally, Trypanosomatidae sp. isolates P041 and P116 from both the arid and humid zones in Santa Cruz and P247 from the mangrove site on Isabela also appeared identical to each other. Given this, only one distinct sequence from each group was used in the phylogenetic analysis, shown in Figure 2.

Evolutionary nucleotide differences between sequences are shown in Table 2. Pairwise sequence divergence between our acquired parasite sequences ranged from 0.11% to 1.08% base pair differences. Given nucleotide differences and the phylogenetic tree estimate (Figure 2), all parasites in *P. downsi* were most closely related to insect-specific trypanosomatids, specifically from the genera *Crithidia* and *Blastocrithidia*. Trypanosomatidae sp. P120 most closely grouped with *Blastocrithidia miridarum*, with high support from both ML bootstrap and Bayesian posterior probabilities and a 0.21% sequence divergence. Additionally, Trypanosomatidae sp. P041 most closely grouped with *Crithidia bombi* with 0.32% sequence divergence; however, support was low for this node in the phylogenetic estimate. Trypanosomatidae sp. isolates P034 and P057 both grouped closest to *Crithidia confusa*, indicating probable identification for both as *Crithidia* species.

## 4. Discussion

Our study detected a high prevalence of insect-specific monoxenous trypanosomatids in *P*. *downsi* collected from different locations in the Galapagos archipelago; to our knowledge this is the first report of trypanosomatids associated with the genus *Philornis*. The high prevalence and wide distribution of *P. downsi* with trypanosomatids suggests that either *P. downsi* entered Galapagos with these parasites or, alternatively, that this is a “new” association of *P*. *downsi* with trypanosomatids found already in the Galapagos that have successfully adapted to this introduced species. If the first possibility applies, it raises concern of infection of other insect species in Galapagos with perhaps the greatest risk to species in the high elevation areas where infection was found to be highest.

The trypanosomatids found in our study were most closely related to the insect-specific genera *Crithidia* and *Blastocrithidia,* though both morphological and genetic studies are required to confirm their identification; taxonomic methods cannot be relied on exclusively to identify trypanosomatids [21]. To our knowledge, this is the first time that any of these trypanosomatid genera have been recorded in insects in the Galapagos Islands.

Both *Crithidia* and *Blastocrithidia* can be pathogenic, depending on the insect host and the parasite species [56]. For example, *Crithidia bombi* has been reported to have negative effects on infected bumblebees, including loss of body mass and decreased fitness [30]. Additionally, although studies have shown that *C*. *fasciculata* infections in mosquitoes are generally non-pathogenic, infections of *C*. *fasciculata* in tsetse flies (*Glossina* spp.) caused rapid death of the flies within nine days [31]. Some species of *Blastocrithidia*, a closely related genus, can also have negative effects on their hosts including decreased foraging activity [32], decreased survivorship and delayed developmental processes [29], changes in digestive processes and reproduction and decreased immune system functions [33], and multiple population collapses [34].

Given the evidence of both vertical and horizontal transmission of the genera *Crithidia* and *Blastocrithidia* in insects [24,25,26,27], either mode of transmission is likely between conspecifics of *P. downsi*; however, only horizontal transmission would be the expected transmission route to other insect species. Between conspecifics, vertical transmission could occur if eggs of *P. downsi* are contaminated with adult feces containing parasites, and larvae acquire these parasites as they emerge from eggs, or if the nest-inhabiting larvae come into contact with the feces of infected adult *P*. *downsi* in the nest material. On the other hand, horizontal transmission could be possible during the adult stage via ingestion of infected insect fecal matter or via an infected food source such as fruits [28]. Although, the immature stages of *P. downsi* are blood feeders, adults are plant feeders and are known to feed on a variety of endemic and introduced fruits in the Galapagos Islands [16]. The broad range of plant species visited by *P. downsi* could also facilitate the exchange of these trypanosomatids with other insects found in Galapagos.

## 5. Conclusions

Our study provides the first evidence of infection of *Philornis* species with insect-specific trypanosomatids. Neither of the most closely genetically related genera detected in *P. downsi*, *Crithidia* and *Blastocrithidia*, have previously been reported in the Galapagos Islands. Molecular screening of *P*. *downsi* from mainland Ecuador and of other insects (in particular mosquitoes and other Diptera) near the sites where we collected infected *P*. *downsi* in the Galapagos Islands will help determine the source of these trypanosomatids. Irrespective of whether these parasites arrived with *P*. *downsi* or have been acquired since the fly’s introduction to the Galapagos Islands, its successful colonization and establishment and its wide-reaching impacts on avian species in the Galapagos Islands suggest that these trypanosomatids are not causing substantial pathogenic effects on *P*. *downsi*. The next step is to determine whether *P*. *downsi* poses an additional challenge to the conservation of the unique fauna of the Galapagos archipelago as a vector of insect parasites.

## Figures and Tables

**Table 1:** Number of samples tested for trypanosomatids from locations on Santa Cruz and Isabela islands; number of sample pools that tested positive using primers from [44,45] and MIR, minimum infection rate (the number of pools testing positive for trypanosomatids/total number of flies tested) using primers from [44].

Island	Site	Total Sample Pools (# Total Flies)	Molecular Tests (# Positive Pools/# Total Tested Pools)		MIR (%)
			[44] ^1^	[45] ^1^	
Santa Cruz	Lowland, dry (El Barranco)	165 (376)	138/165	19/40	36.7
	Agricultural zone (Los Guayabillos)	2 (2)	2/2	0/0	100.0
	Highland, humid (Los Gemelos)	110 (267)	109/110	18/43	40.8
Isabela	Mangrove forest (Playa Tortuga Negra)	17 (45)	14/17	3/9	31.1
	Lava field (Playa Tortuga Negra)	3 (4)	3/3	0/3	75.0
	Total	297 (694)	267/297	40/95	38.5

^1^ Indicates primer source for each test.

**Table 2:** Estimates of evolutionary divergence comparing sequences obtained from *Philornis downsi* and known GenBank reference sequences. Partial sequences from the conserved region of the 18S rRNA gene were analyzed. Percent differences of base substitutions between sequences are shown below the diagonal. Standard error estimates are shown above the diagonal. Analyses were conducted using the Jukes–Cantor model [55]. The rate variation among sites was modeled with a gamma distribution (shape parameter = 1). The analysis involved 17 nucleotide sequences. All positions with less than 95% site coverage were eliminated. That is, fewer than 5% alignment gaps, missing data, and ambiguous bases were allowed at any position. There were a total of 938 positions in the final dataset. Evolutionary analyses were conducted in MEGA6 [49]. All GenBank accession numbers and strain/isolate names can be found in Table S3.

		1	2	3	4	5	6	7	8	9	10	11	12	13	14	15	16	17
1	Trypanosomatidae sp. P041		0.17	0.14	0.30	0.32	0.19	0.29	0.43	1.58	1.44	1.58	1.35	1.41	1.44	1.54	1.48	1.46
2	Trypanosomatidae sp. P034	0.32		0.11	0.32	0.34	0.26	0.34	0.49	1.53	1.42	1.53	1.34	1.41	1.41	1.51	1.44	1.43
3	Trypanosomatidae sp. P057	0.21	0.11		0.34	0.35	0.24	0.32	0.46	1.53	1.42	1.53	1.34	1.41	1.41	1.51	1.44	1.42
4	Trypanosomatidae sp. P120	0.86	0.97	1.08		0.21	0.37	0.41	0.50	1.57	1.40	1.57	1.32	1.37	1.45	1.52	1.48	1.47
5	*Blastocrithidia miridarum*	0.97	1.08	1.19	0.43		0.39	0.42	0.54	1.57	1.40	1.57	1.31	1.38	1.43	1.53	1.47	1.46
6	*Crithidia bombi*	0.32	0.65	0.54	1.19	1.30		0.36	0.50	1.57	1.45	1.57	1.35	1.42	1.46	1.54	1.50	1.48
7	*Crithidia confusa*	0.75	1.08	0.97	1.52	1.63	1.08		0.53	1.59	1.43	1.59	1.34	1.37	1.41	1.51	1.44	1.42
8	*Crithidia dedva*	1.75	2.08	1.97	2.19	2.53	2.08	2.42		1.54	1.43	1.54	1.37	1.47	1.41	1.46	1.46	1.47
9	*Leptomonas collosoma*	15.12	14.81	14.81	15.12	15.27	14.81	15.12	14.96		1.56	0.15	1.50	1.50	1.48	1.52	1.43	1.40
10	*Leptomonas mirabilis*	14.20	14.05	14.05	13.75	14.05	14.50	14.20	14.35	16.52		1.55	1.10	1.11	1.42	1.40	1.42	1.41
11	*Leptomonas rigidus*	15.12	14.81	14.81	15.12	15.27	14.81	15.12	14.81	0.21	16.36		1.49	1.49	1.48	1.52	1.43	1.40
12	*Leptomonas samueli*	13.16	13.01	13.01	12.86	12.86	13.16	13.31	13.75	15.74	9.49	15.58		0.80	1.54	1.62	1.55	1.55
13	*Leptomonas costoris*	14.50	14.50	14.50	13.90	14.20	14.50	14.20	15.27	15.89	9.49	15.74	5.61		1.49	1.53	1.47	1.46
14	*Trypanosoma avium*	13.90	13.75	13.60	13.90	13.75	14.20	13.45	13.75	14.96	15.42	14.96	16.05	15.74		0.80	0.63	0.62
15	*Trypanosoma bennetti*	15.12	14.96	14.96	14.81	14.96	15.12	14.66	14.66	15.58	14.96	15.58	16.84	16.52	5.37		0.82	0.84
16	*Trypanosoma corvi*	14.50	14.35	14.20	14.50	14.35	14.81	13.90	14.35	14.50	15.74	14.50	16.52	16.36	3.93	5.49		0.18
17	*Trypanosoma culicavium*	14.20	14.05	13.90	14.20	14.05	14.50	13.60	14.50	14.05	15.74	14.05	16.52	16.36	4.04	5.86	0.32	

## Supplementary Materials

The following are available online at https://www.mdpi.com/2075-4450/11/7/422/s1, Table S1: Recipes for Trypanosomatidae PCR tests, Table S2: 18S rRNA gene sequences from *P. downsi* samples generated during this study, Table S3: 18S rRNA gene sequences used in phylogenetic analyses, including sequences obtained from *P. downsi* samples from our study and known reference sequences from GenBank.
