# Supplementary material for: Trypanosomatids Detected in the Invasive Avian Parasite Philornis downsi (Diptera: Muscidae) in the Galapagos Islands
Source: Insects. 2020 Jul 9;11(7):422. doi: 10.3390/insects11070422 (PMC7411904; doi:10.3390/insects11070422)
Supplement: Supplementary file 1 [file insects-11-00422-s001.zip › Supplementary Tables.docx]

**Table S1.** Recipes for Trypanosomatidae PCR tests. References after primers indicate primer sources.

| **PCR component** | **Concentration** | **Primers [44]** | | **Primers [45]** |
| --- | --- | --- | --- | --- |
|  |  | *Outer (µl)* | *Inner (µl)* | *(µl)* |
| Ex Taq ™ Buffer | 10X | 2.5 | 2.5 | 2.5 |
| DNTPs | 2.5 mM/ each  (Total 10 mM) | 2.0 | 2.0 | 2.0 |
| MgCl_2_ | 25 mM | 2.0 | 2.0 | 2.0 |
| Primer 1 | 10 uM | 1.0 | 1.0 | 1.0 |
| Primer 2 | 10 uM | 1.0 | 1.0 | 1.0 |
| BSA | - | 0.3 | 0.3 | - |
| ddH_2_0 | - | 15.0 | 15.0 | 14.3 |
| TaKaRa Ex Taq | - | 0.2 | 0.2 | 0.2 |
| DNA/Amplicon | - | 1.0 (DNA) | 1.0 (Amplicon) | 2.0 (DNA) |
| Total |  | 25 | 25 | 25 |

**Table S2.** 18S rRNA gene sequences from *P.* *downsi* samples generated during this study. All sequences were deposited in GenBank with the corresponding accession numbers.

| **Species/strain** | **GenBank accession number** | **Host organism** |
| --- | --- | --- |
| Trypanosomatidae sp. P034 | MG787532 | *P*. *downsi* (Nest fly) |
| Trypanosomatidae sp. P041 | MG787533 | *P*. *downsi* (Nest fly) |
| Trypanosomatidae sp. P057 | MG787534 | *P*. *downsi* (Nest fly) |
| Trypanosomatidae sp. P091 | MG787535 | *P*. *downsi* (Nest fly) |
| Trypanosomatidae sp. P116 | MG787536 | *P*. *downsi* (Nest fly) |
| Trypanosomatidae sp. P120 | MG787537 | *P*. *downsi* (Nest fly) |
| Trypanosomatidae sp. P129 | MG787538 | *P*. *downsi* (Nest fly) |
| Trypanosomatidae sp. P247 | MG787539 | *P*. *downsi* (Nest fly) |
| Trypanosomatidae sp. P322 | MG787540 | *P*. *downsi* (Nest fly) |
| Trypanosomatidae sp. P361 | MG787541 | *P*. *downsi* (Nest fly) |
|  |  |  |

**Table S3.** 18S rRNA gene sequences used in phylogenetic analyses, including sequences obtained from *P*. *downsi* samples from our study and known reference sequences from GenBank.

| **Species/strain** | **GenBank accession number** | **Host organism (Common name)** |
| --- | --- | --- |
| Trypanosomatidae sp. P034 ¹ | MG787532 | *P*. *downsi* (Nest fly) |
| Trypanosomatidae sp. P041 ¹ | MG787533 | *P*. *downsi* (Nest fly) |
| Trypanosomatidae sp. P057 ¹ | MG787534 | *P*. *downsi* (Nest fly) |
| Trypanosomatidae sp. P120 ¹ | MG787537 | *P*. *downsi* (Nest fly) |
| *Blastocrithidia miridarum* ZM | EU079128.1 | *Zelurus martinsi* (Reduviid bug) |
| *Crithidia bombi* beb1 | FN546181.1 | *Bombus terrestris* (Bumblebee) |
| *Crithidia confusa* isolate 320AR | JF717837.1 | *Largus maculatus* (True bug) |
| *Crithidia dedva* strain D2 | JN624299.1 | *Nabis flavomarginatus* (Damsel bug) |
| *Leptomonas mirabilis* isolate TCC301E | JQ359729.1 | *Cynomya cadaverina* (Bottle fly) |
| *Leptomonas costoris* | JQ359728.1 | *Gerris comatus* (Water strider) |
| *Leptomonas samueli* | JQ359722.1 | *Zelus leucogrammus* (Assassin bug) |
| *Leptomonas rigidus* | JN582049.1 | *Salda littoralis* (Shore bug) |
| *Leptomonas collosoma* | JN582046.1 | *Gerris dissortis* (Water strider) |
| *Trypanosoma corvi* BUT17 | JN006854.1 | *Buteo buteo* (Buzzard) |
| *Trypanosoma culicavium* PAS99 | HQ107969.1 | *Ficedula albicollis* (Collared flycatcher) |
| *Trypanosoma avium* ABUT/CZ/99/BUT15 | AY099320.2 | *Buteo buteo* (Buzzard) |
| *Trypanosoma benneti* APO7 | JF778738.1 | *Aquila pomarina* (Eagle) |

¹ Sequences from *Philornis* *downsi* samples generated during this study.
